# In search of blue-light effects on cognitive control

**DOI:** 10.1038/s41598-021-94989-6

**Published:** 2021-07-29

**Authors:** Hsing-Hao Lee, Yun-Chen Tu, Su-Ling Yeh

**Affiliations:** 1 Department of Psychology, National Taiwan University, Taipei, Taiwan; 2 Graduate Institute of Brain and Mind Sciences, National Taiwan University, Taipei, Taiwan; 3 Neurobiology and Cognitive Science Center, National Taiwan University, Taipei, Taiwan; 4 Center for Artificial Intelligence and Advanced Robotics, National Taiwan University, Taipei, Taiwan

**Keywords:** Psychology, Human behaviour

## Abstract

People are constantly exposed to blue light while engaging in work. It is thus crucial to understand if vast exposure to blue light influences cognitive control, which is essential for working efficiently. Previous studies proposed that the stimulation of intrinsically photosensitive retinal ganglion cells (ipRGCs), a newly discovered photoreceptor that is highly sensitive to blue light, could modulate non-image forming functions. Despite studies that showed blue light (or ipRGCs) enhances brain activations in regions related to cognitive control, how exposure to blue light changes our cognitive control behaviorally remains elusive. We examined whether blue light influences cognitive control through three behavioral tasks in three studies: the sustained attention to response task (SART), the task-switching paradigm, and the Stroop task. Classic effects of the SART, switch cost, and the Stroop effect were found, but no differences were observed in results of different background lights across the six experiments. Together, we conclude that these domains of cognitive control are not influenced by blue light and ipRGCs, and whether the enhancement of blue light on brain activities extends to the behavioral level should be carefully re-examined.

## Introduction

People nowadays are exposed to blue light constantly through cellphones, laptops, and tablets, and thus it has become critical to understand whether and how the high-dosages of blue-light exposure affect human cognition. Recently a newly discovered photoreceptor type distinct from the classic S, M, and L cones—the intrinsically photosensitive retinal ganglion cells (ipRGCs)^1^—was found to be mainly in charge of non-image forming functions. ipRGCs express melanopsin which is most sensitive to the light in the spectrum of 460 to 480 nm, the wavelength range of blue light. ipRGCs are also shown to be involved in non-visual functions, such as photoentrainment circadian rhythm, pupillary reflex, and alertness^2^. However, it remains unclear whether blue light enhances or impairs cognitive control, which allows us to focus on the current goal while working. If blue light can facilitate cognitive control, working in a blue-light enriched environment would be more effective than other lighting conditions. Hence, examining the effects of blue light on cognitive control can further help designers or managers decide the lighting environment for enhancing work efficiency.

However, despite some hints from brain activations for networks related to cognitive control, behavioral evidence for blue-light effects on cognitive control is still lacking. For example, Vandewalle and colleagues have shown that blue light activates brain regions related to executive control during a two-back working memory task: Stronger activations were found in the medial prefrontal cortex (mPFC), anterior cingulate cortex (ACC), inferior frontal cortex (IFC), and thalamus under blue light compared to a variety of control conditions, such as background green light^3,4^, violet light^3^, orange light^5^, and darkness^6^. Even though the authors proposed that blue light might facilitate working memory, only one of these studies^5^ provided evidence that the behavioral performance in the two-back working memory task was indeed better than that of the control light condition.

Despite that the modulation of activity in the cortical areas usually affects behavior, this does not apply to every situation. As the global neuronal workspace theory states, conscious behavioral responses through, say, perception and cognition, are associated with increased activity in the cerebral cortex. Therefore, the conscious experience follows an all-or-none manner: conscious experience can only occur after workspace neurons have activated to a certain extent^7,8^. This theory can be applied to the findings of blue light and neuroimaging studies and a relevant question naturally comes to mind: can the activated brain response induced by blue light extend to the behavioral level? The main goal of the current study is to directly examine if blue light can influence cognitive control at the behavioral level.

Cognitive control refers to a family of top-down processes that allow us to concentrate on our current work while inhibiting irrelevant stimuli^9,10^, which affects career success, mental health, and quality of life^9^. There are three core parts of cognitive control^11^: inhibition, working memory, and cognitive flexibility. Inhibition (or sometimes referred to as inhibitory control) includes three aspects of inhibitory functions that are crucial to preventing interference in the ongoing task: inhibiting thoughts (cognitive inhibition), suppressing irrelevant features and focusing on relevant ones (selective attention), and inhibiting unwanted actions (response inhibition). Working memory involves holding and performing mental operations on information in the mind, which allows us to incorporate new information while keeping the goal in mind (updating). Cognitive flexibility refers to the ability to shift from one mental set to another. Despite the distinct definitions of the three components in cognitive control, they are tightly related. For example, inhibitory control helps clear out the mental workspace of irrelevant thoughts for working memory operation^9^. Cognitive flexibility involves both inhibitory control and working memory to operate, and is highly related to these two constructs. For example, switching back and forth between different mental sets requires inhibiting the irrelevant mental set (inhibitory control) while activating the ongoing one (working memory)^9^. Together, these functions allow us to exert top-down control, which helps maintain the current goal and prevent interferences.

Previous studies showed that the inhibition of unwanted actions was not enhanced under blue light. Cajochen et al.^12^ and Chellappa et al.^13^ used LED lights as the source of blue light and the go/no-go task to examine if alertness and motor inhibition can be enhanced under blue light. Despite that the facilitation of reaction time was found in both studies, the no-go error rate, which serves as the index of response control^14^, did not decrease under blue light. As the go/no-go task measures the ability to inhibit unwanted actions, it is unknown if the inhibition of irrelevant thoughts and visual input, different domains of inhibition, are influenced by blue light, which is what we aimed to test in the current study.

Ferlazzo et al.^15^ showed the very first behavioral evidence that blue light could facilitate cognitive flexibility, but their study was not able to probe into which, if any, components (S and ipRGCs) of light facilitate cognitive control. They used a 4000 K LED light as light source and found that blue light decreased switch cost (the reaction time of switching mental sets subtracted from the RT of the repeated mental set) in a task-switching paradigm. However, they used a halogen light (2800 K) as the control light, thus, luminance and the S, M, and L cones’ activations would have been very different from the ones in the LED light conditions. As a result, the facilitatory effect in their study could have been driven by differences in luminance rather than S cones or ipRGCs, namely, blue light per se.

The generalizability in other studies which showed facilitated cognitive flexibility under blue light was limited, as the effects of blue light were tested only during the afternoon dip. For example, Slama et al.^16^ compared switch cost across light conditions with white light enriched blue light in the range of 460 nm and white light enriched orange light in the range of 600 nm during the afternoon session, which aimed to compare the improving effect of a nap to bright light exposure. They found that the participants who were exposed to blue light showed smaller switch costs compared to the nap group, suggesting that blue light is a better intervention for the afternoon dip. However, Kaida et al.^17^ provided the opposite evidence and showed smaller switch costs in the nap group compared to the blue light-exposure (7000–7500 K) group. Consequently, previous studies showed inconsistent results on the effects of blue light on cognitive flexibility.

Given previous studies’ inconsistent results regarding blue-light effects on cognitive control and the lack of decent control conditions, the current study adopted well-controlled background lights and manipulation of activation in ipRGC level to help resolve the discrepancy. We aimed to unveil whether blue light can influence cognitive control from three different tasks: In Study 1, the sustained attention to response task (SART)^18–20^ was used to examine inhibition of irrelevant thoughts, in Study 2, the task switching paradigm (adapted from Rogers and Monsell^21^ and Yeh et al.^22^) was used to measure cognitive flexibility, and in Study 3, the Stroop task^23^ was used to examine the inhibition of irrelevant visual features. Both the SART and Stroop task were used to examine if blue light influences inhibitory control, but as the former is more related to irrelevant thought inhibition^18^ and the latter is more related to irrelevant visual stimuli inhibition^9^, we also aimed to test both aspects of inhibition. In addition, the task switching paradigm is highly associated with the Stroop task^24,25^ as they share similar neural mechanisms^26^, and has been widely used to investigate cognitive flexibility^9^. Therefore, we considered the task switching paradigm to be adequate to examine if cognitive flexibility, which is also highly associated with inhibitory control and working memory, is influenced by blue light. By manipulating the background colors of the monitor to blue or orange (with similar luminance) and blue-light filtered or unfiltered conditions, we aimed to test if blue light and ipRGCs affect cognitive control. Across the six experiments, we provided Bayesian Factors calculated in JASP (http://www.jasp-stats.org) to verify whether our results were in favor of the null or alternative hypothesis. We used the default prior (*r* = 0.707) and Cauchy distribution (centered at zero) in the JASP to calculate the Bayesian Factors^27^. A *BF*_*10*_ less than 0.33 indicates data favoring the null hypothesis, and a *BF*_*10*_ greater than 3.00 indicates data favoring the alternative hypothesis, while a *BF*_*10*_ between 0.33 and 3.00 indicates an inconclusive result^27–29^.

## Study 1

In Study 1, we aimed to investigate if the ability to inhibit irrelevant thoughts would be influenced by blue light. We used the SART to measure this construct, which is an adapted go/no-go task to investigate the extent to which we can inhibit mind-wandering (MW). The dependent variables in this task were self-report MW rate, no-go error rate (commission error rate), reaction time coefficient of variability (RTCV, the standard deviation of reaction time and the mean reaction time ratio), reaction time (RT), anticipation rate (RT faster than 100 ms), and omission rate (no response to the go stimulus). In Experiment 1, blue and orange light backgrounds with similar luminance were manipulated through the monitor. Furthermore, in Experiment 2, we used a filtered glass (compared to a transparent glass, see “Methods”) to directly investigate if ipRGCs play a role in affecting the inhibition of irrelevant thoughts. If blue light or ipRGCs enhance(s) irrelevant thoughts inhibition, we should observe lower MW rates, lower commission error rates, lower RTCV, faster RTs, lower anticipation rates, and less omission rates in the blue light condition and unfiltered condition compared to the orange light condition and filtered condition, respectively. However, if blue light and/or ipRGCs do not contribute to the inhibition of irrelevant thoughts, no differences across these indices should be found across light conditions.

### Experiment 1

#### Methods

##### Participants

Our sample size was based on the effect size of Experiment 3 in Chen and Yeh^30^ (Cohen’s *f* = 0.42), which examined the effects of blue light (compared to orange light) on dynamic vision. We calculated our sample size based on Chen and Yeh^30^, given that dynamic vision^31^ and cognitive control^32^ both require attentional resources, and Chen and Yeh^30^ is the first study to use a blue-light filter^33^ to investigate the influence of blue light on cognitive functions. To reach adequate statistical power (0.8), we needed 14 participants according to G*Power 3.0^34^. To be more conservative, we recruited relatively more participants (50% additional) than theoretically needed to ensure that we have sufficient power to detect the effect (if any) of blue light on different conditions. Hence, twenty-four participants (age range: 19–40, 13 males) who were free from psychological and neuronal disorders were recruited. Our sample size is relatively larger than that of other studies investigating blue-light effects on working memory (e.g., N = 14 in Daneault et al.^5^; N = 18 in Vandewalle et al.^3^; N = 15 in Vandewalle et al.^4^), hence, statistical power in our experiment(s) should be sufficient. Participants had normal or corrected-to-normal vision, and did not wear any blue-light-filtering products. Participants gave informed consent before the experiment and received extra course credit (1.0 point) or $160 NTD (approximately $5.5 USD) for their participation of a 1-h experiment. This study was approved by the Research Ethics Committee at National Taiwan University and implemented in compliance to the guidelines and regulations.

##### Apparatus and stimuli

**Figure 1:**
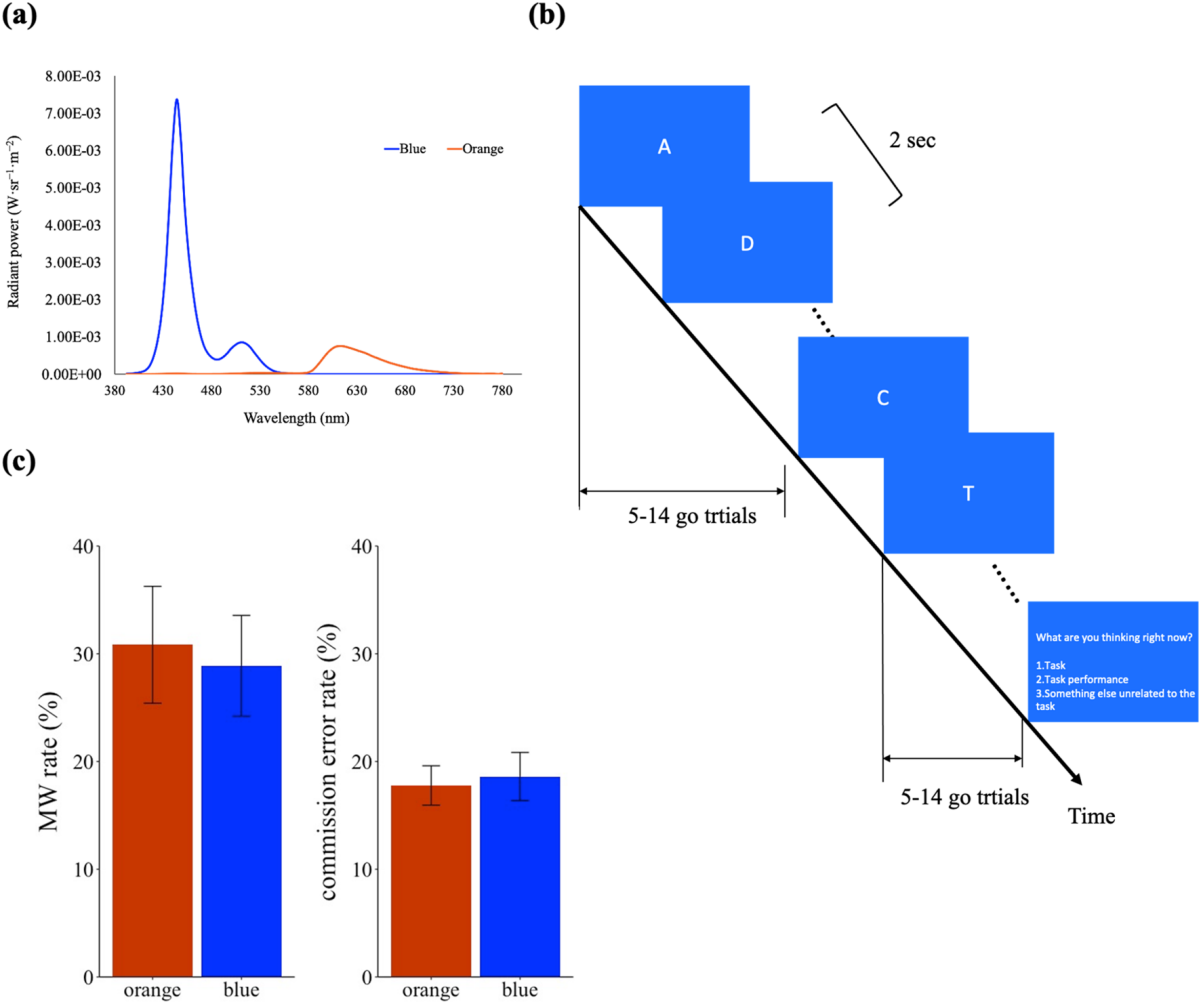
(**a**) The color spectra of the blue and orange backgrounds in Experiments 1 and 3. (**b**) The experimental design of Experiment 1. (**c**) Mind-wandering (MW) rate and commission error rate in Experiment 1. Error bars represent one S.E.M.

Participants were seated in a completely dark room with the monitor as the only light source. Participants were instructed to place their head on a chin-rest, which was 57 cm from the monitor. The light conditions and stimuli were manipulated through the monitor using MATLAB (The MathWorks), and the light conditions were the same as the conditions in Experiment 1A of Chien et al.^35^ We chose orange light as the control condition given that its wavelength overlaps the least with blue light so the contribution of different cones and ipRGCs can be separated more easily^30,36^. The English letters were presented in white (201.79 cd/m^2^) against the blue (luminance: 16.59 cd/m^2^, CIE: 0.152, 0.063) or orange (luminance: 16.75 cd/m^2^, CIE: 0.631, 0.355) background on the monitor. Figure 1a and the upper part of Table 1 show the color spectra and the stimulations of each photoreceptor in both backgrounds. Twenty English letters (A, B, C, D, E, F, G, H, I, J, K, L, M, O, P, Q, R, S, T, U) were chosen as stimuli and extended 1.56°, presented in Calibri font at the center of the screen.

**Table 1:** Stimulation of cones and ipRGCs in the experiments.

Experiment	Background	L	M	S	ipRGC
Experiments 1 and 3	Blue	2.65	2.03	42.64	19.43
	Orange	2.67	0.46	0.15	0.28
	Ratio (blue/orange)	0.99	4.41	284.27	69.39
Experiments 2 and 4	Unfiltered	8.30	3.62	13.63	11.55
	Filtered	8.57	3.70	12.03	8.09
	Ratio (blue/orange)	0.97	0.98	1.13	1.43

##### Design and procedure

Before the experiment began, participants were instructed to stay in a dark room for 5 min for dark adaptation to equate participants’ experiences towards external light. Half of the participants experienced the blue light condition first followed by another 5-min dark adaptation and then the orange light condition; the other half of the participants experienced the reverse order. Participants practiced the SART under either the blue or orange background, and the order of light being administered was counterbalanced across participants. The experimental design is shown in Fig. 1b. Participants were instructed to press the space button as quickly as possible whenever they saw an English letter on the screen (i.e., go trials). However, they were instructed to withhold their response whenever they see the no-go target, which was the English letter “C”. The no-go target appeared pseudorandomly across the experiment (i.e., no-go trials). Each letter was presented for 1000 ms or until the participant responded. The inter-trial interval (ITI) varied with the reaction time of the participants so that each trial (including the ITI) lasted for 2000 ms. Probe questions were pseudorandomly assigned in the experiment to ask participants “What are you thinking right now?” with the following three options: (1) Task, (2) Task performance, and (3) Something else unrelated to the task^19^, which corresponded to the buttons on the number-pad 1, 2, and 3. Participants were told to respond within 5 s and that there was no correct answer regarding this question. No-go targets and probes were inserted into go trials separately every 5–14 trials, so that they were distributed relatively evenly across the experiment. For each light condition, there were 285 go trials (95% among all trials) with 15 no-go trials (5% among all trials) and 15 probes in the formal experiment. For the practice trials, there were 80 go trials with 5 probes and 5 no-go targets. Participants usually finished one light exposure condition in around 30 min, which was a similar duration as our previous studies^30,36^. We believe this duration for light exposure is appropriate given that our previous studies^30,36^ also had similar durations and we observed a significant facilitation of blue light in dynamic vision and saccade latency.

#### Results

We analyzed the following six indices as the performance in the SART across light conditions: (1) MW rate (subjective report to option 3 or no response to the probe), (2) commission error rate, (3) RTCV, (4) RT, (5) anticipation rate, and (6) omission rate. Figure 1c shows the results of MW rate and commission error rate. None of the indices showed significant differences across light conditions (MW rate: *t*(23) = − 0.43, *p* = 0.669, *BF*_*10*_ = 0.234; commission error rate: *t*(23) = − 0.43, *p* = 0.671, *BF*_*10*_ = 0.234; RTCV: *t*(23) = − 0.75, *p* = 0.458, *BF*_*10*_ = 0.278; RT: *t*(23) = − 0.88, *p* = 0.386, *BF*_*10*_ = 0.305; anticipation rate: *t*(23) = − 0.64, *p* = 0.529, *BF*_*10*_ = 0.258; omission rate: *t*(23) = − 1.42, *p* = 0.168, *BF*_*10*_ = 0.523).

We also analyzed the RT and RTCV before the no-go target (i.e., C) to verify the validity of the SART. We compared the performance between the five go-trials before the no-go target of failed-to-stop and successful-stop trials^18,37^. Significant slower RTs (*t*(23) = 17.44, *p* < 0.001, *BF*_*10*_ = 6.32 × 10^11^) and smaller RTCV (*t*(23) = − 10.45, *p* < 0.001, *BF*_*10*_ = 3.12 × 10^7^) were found in the successful-stop trials (mean RT = 395.35 ms, mean RTCV = 0.15) compared to the ones in the failed-to-stop trials (mean RT = 339.37 ms, mean RTCV = 0.17), which were in line with previous findings and ensured the validity of the current experiment^18,37^.

### Experiment 2

#### Methods

**Figure 2:**
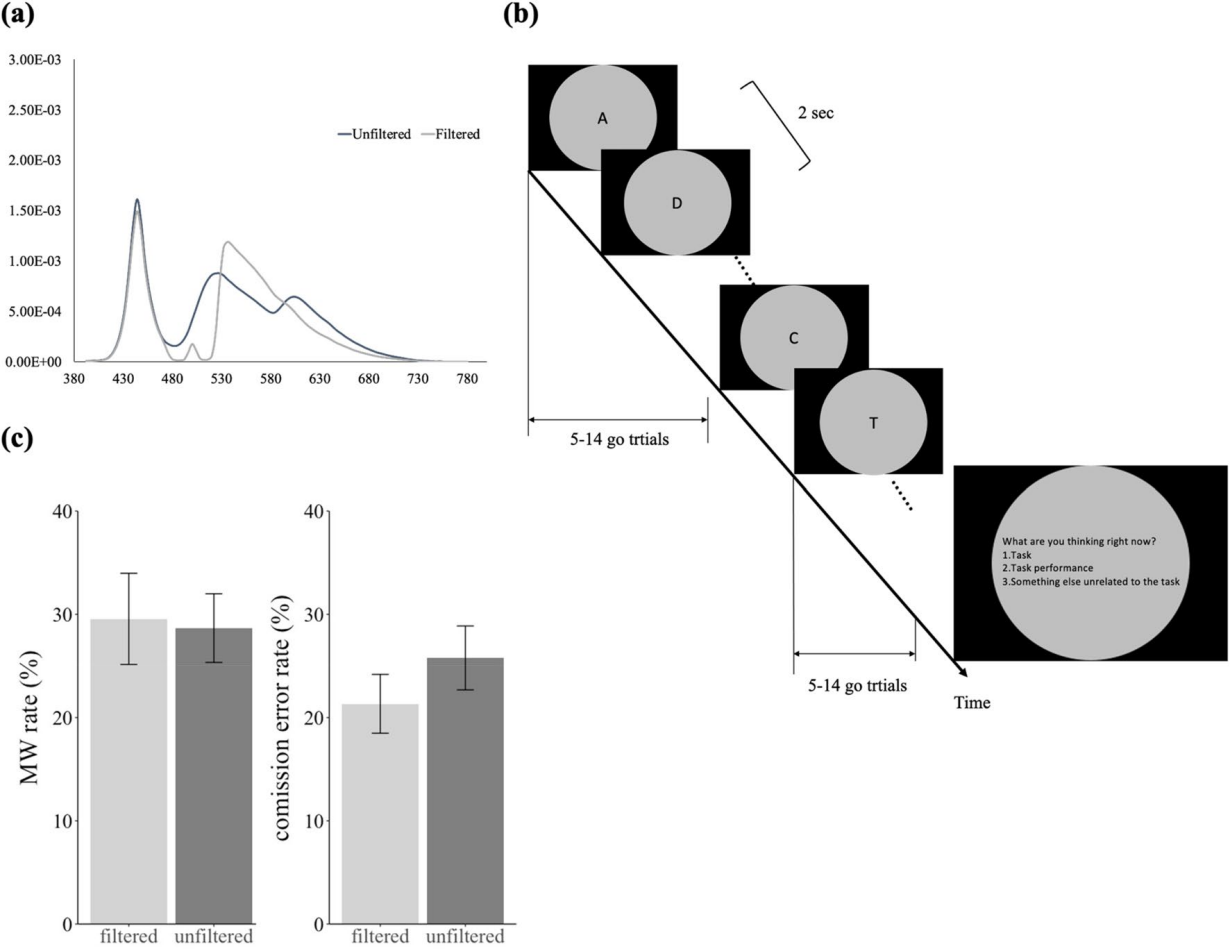
(**a**) The color spectra of the blue and orange backgrounds in Experiments 2 and 4. (**b**) The experimental design of Experiment 2. (**c**) Mind-wandering (MW) rate and commission error rate in Experiment 2. Error bars represent one S.E.M.

A new group of 30 participants (age range: 18–27, 14 males) were recruited in the current experiment. The criteria for recruiting participants and most of the experimental settings were the same as those of Experiment 1 apart from the following detailed below. First, we used a blue-light filter to reduce the intensity of light with wavelengths between 450 to 525 nm by half^33^, to directly test if ipRGCs contribute to cognitive inhibition, while compensating for the colors that were blocked. Hence, the background colors across all conditions were grey (metamer) and similar in luminance (unfiltered condition: luminance: 45.33 cd/m^2^, CIE: 0.311, 0.336; filtered condition: luminance: 47.16 cd/m^2^, CIE: 0.325, 0.359), which is the same as the Experiment 2B of Chien et al.^35^. Figure 2a and the lower part of Table 1 show the color spectra and the stimulations of each photoreceptor in both backgrounds. Second, participants were instructed to look at the monitor through the filtered (filtered condition) or transparent lens (unfiltered condition) with only their right eye while the left eye is covered by a piece of black glass. Half of participants experienced the filtered condition first followed by the unfiltered condition, and the other half of participants experienced the reverse order. Finally, there was a black circle mask (16.48° in radius, matching the scope of the filter) that covered the rest of the screen to help participants focus on the stimuli at the center, and the stimuli were presented in black. The experimental design is shown in Fig. 2b. By comparing the performance between higher ipRGC stimulation (using a transparent lens) and lower ipRGC stimulation (using a filtered lens), we could directly investigate if ipRGCs exert any effect in these domains of cognitive control.

#### Results

We examined the same indices as those of Experiment 1 across light conditions (Fig. 2c). No significant differences across light conditions were found again (MW rate: *t*(29) = − 0.23, *p* = 0.814, *BF*_*10*_ = 0.2; commission error rate: *t*(29) = − 1.65, *p* = 0.111, *BF*_*10*_ = 0.648; RTCV: *t*(29) = − 0.02, *p* = 0.984, *BF*_*10*_ = 0.195; RT: *t*(29) = 0.32, *p* = 0.748, *BF*_*10*_ = 0.204; anticipation rate: *t*(29) = 0.19, *p* = 0.849, *BF*_*10*_ = 0.198; omission rate: *t*(29) = 0.74, *p* = 0.467, *BF*_*10*_ = 0.25).

We also analyzed the RT and RTCV before the no-go target (i.e., C) to verify the validity of the SART, similar to Experiment 1. Significant slower RTs (*t*(29) = 18, *p* < 0.001, *BF*_*10*_ = 1.81 × 10^14^) and smaller RTCV (*t*(29) = − 12.13, *p* < 0.001, *BF*_*10*_ = 1.32 × 10^10^) were found in the successful-stop trials (mean RT = 393.61 ms, mean RTCV = 0.15) compared to the ones in the failed-to-stop trials (mean RT = 350.59 ms, mean RTCV = 0.19).

#### Discussion

In Study 1, we found neither blue-light facilitation nor impairment on inhibition of irrelevant thoughts, which were examined through the MW rate, commission error rate, RTCV, RT, anticipation rate, and omission rate of blue and orange or filtered and unfiltered conditions. To the best of our understanding, these are the first experiments that directly investigated whether blue light can influence thought inhibition. However, with a suitable control of luminance and conservative statistical analyses by providing Bayesian Factors favoring the null hypotheses^28^ across the six indices, we found that neither exposure to blue light nor the stimulation of ipRGCs influences the ability to inhibit irrelevant thoughts.

## Study 2

In Study 2, we aimed to investigate whether cognitive flexibility would be influenced by blue light. As previous studies provided positive evidence regarding blue-light effects on task switching ability^15,16^, similar results should be observed in our experiments while controlling for luminance across background colors. We used the adapted version of the alternating-runs procedure in a task-switching paradigm^21,22^. Switch cost served as the dependent variable in the experiment, which was calculated as the RT of the switching mental set subtracted by the RT of the repeated mental set^24,25^. In Experiment 3, participants were exposed to blue and orange light, and in Experiment 4, another group of participants were exposed to filtered and unfiltered light. Smaller switch costs in the blue light condition and unfiltered condition (compared to the orange light condition and filtered condition) should be observed if blue light and/or ipRGCs enhance cognitive flexibility. Otherwise, no differences across switch costs should be found across light conditions.

### Experiment 3

#### Methods

##### Participants

A different group of 32 participants (age range: 19–25, 14 males) were recruited in the current experiment. Other criteria were the same as in Study 1.

##### Apparatus and stimuli

The monitor and background color were the same as those of Experiment 1. The numbers were presented one by one in one of the four quadrants on the screen, which were each 1.59° from the center. The target stimulus, Arabic numerals extended 2.81° and was presented in white (201.79 cd/m^2^) following the clockwise order starting from quadrant 1. There was a white fixation square (extending 0.78°, 201.79 cd/m^2^) at the center to help participants recognize which quadrant the number was in.

##### Design and procedure

**Figure 3:**
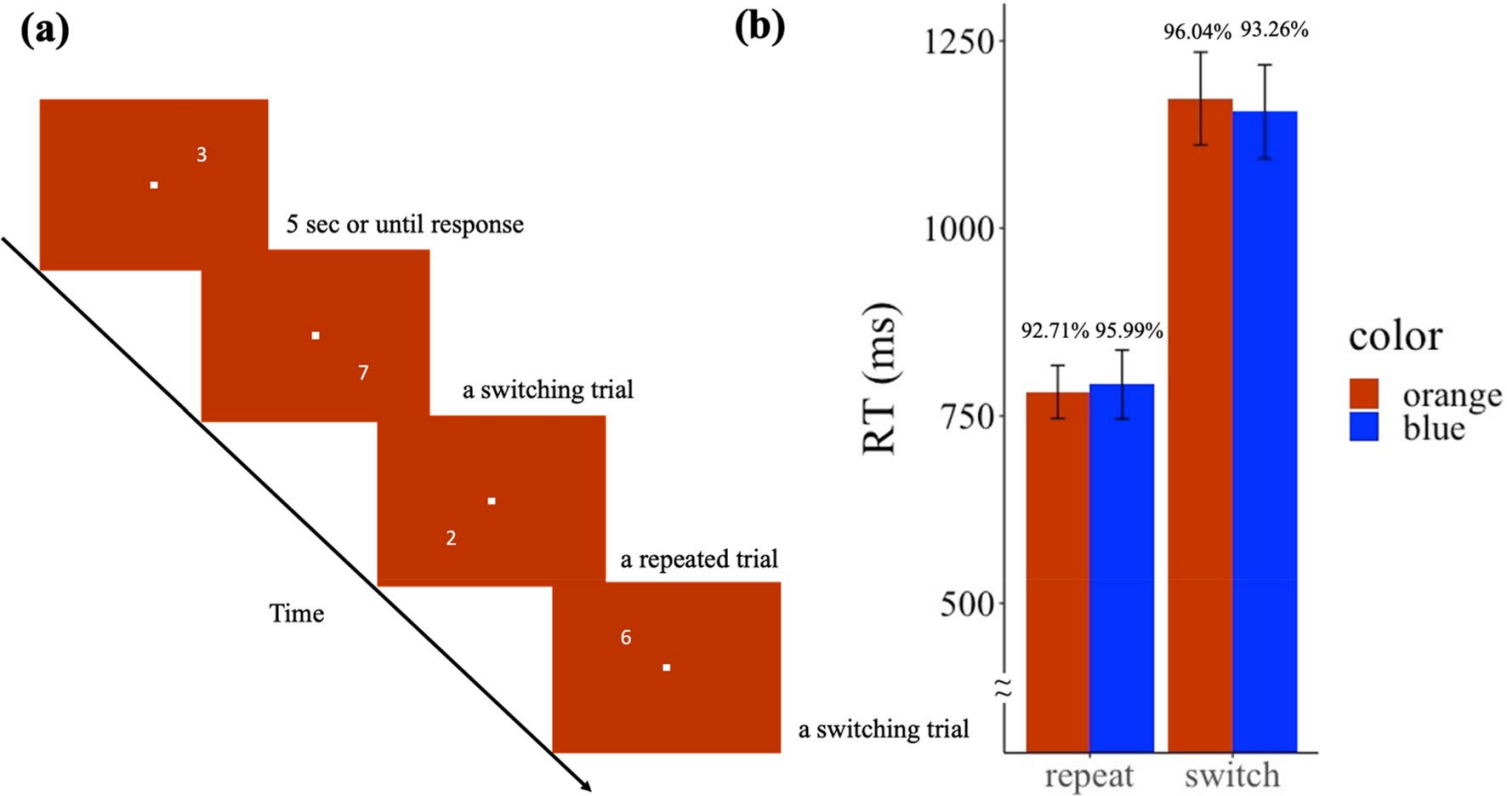
(**a**) The experimental design of Experiment 3. (**b**) Reaction time in repeated and switching trials in Experiment 3. Error bars represent one S.E.M. The number shown above each bar denotes the accuracy of each condition.

The dark adaptation and light conditions procedures were the same as detailed in Experiment 1. After these procedures, participants were instructed to practice the task for 24 trials. The experimental design is shown in Fig. 3a. In this task, the Arabic numerals (1–4, 6–9) would be presented at each quadrant in a clockwise order. Each trial was 5 s or elapsed until participant response. Participants were instructed to judge if the number was odd or even when the number was in the upper visual field (the 1st and the 2nd quadrant), and judge if the number was greater or smaller than 5 if the number was presented in the lower visual field (the 3rd and the 4th quadrant). Participants were instructed to press the “z” button if the number was odd or greater than 5, and press the “/” button if the number was even or smaller than 5. The hand mappings regarding the response were counterbalanced across participants. Participants were instructed to respond as quickly and accurately as possible. For each light condition, there were 120 trials in a block and a total of 2 blocks, while half of the trials were repeated trials and the other were switching trials. Participants were allowed to take a break between the blocks. Participants usually finished one type of light exposure in around 30 min.

#### Results

For the analysis of RT, we removed outlier trials (RT < 300 ms or > 2000 ms), incorrect trials, and the first four trials at the beginning and after the break (which did not belong to either repeated or switching trials). Trials excluded in the analysis were 14.49% of the trials. A two-way repeated-measure analysis of variance (ANOVA) was conducted on the factors of Switch (switching trials, repeated trials) and Light (blue, orange). Figure 3b shows the results. A significant main effect of Switch was found, *F*(1, 31) = 110.9, *p* < 0.001, *BF*_*10*_ = 4 × 10^14^, where the RT to the repeated trials were faster than the RT to the switching trials. However, no effect of Light (*F*(1, 31) = 0.01, *p* = 0.918, *BF*_*10*_ = 0.163) nor interaction (*F*(1, 31) = 0.60, *p* = 0.446, *BF*_*10*_ = 0.174) between Switch and Light were found. The switch cost was not different across light conditions (*t*(31) = 0.77, *p* = 0.446, *BF*_*10*_ = 0.249).

As for the accuracy, the overall accuracy was high (94.5%). A higher accuracy was found in the repeated trials compared to the one in switching trials (*F*(1, 31) = 71.8, *p* < 0.001, *BF*_*10*_ = 3.74 × 10^9^). Neither the main effect of Light (*F*(1, 31) = 0.34, *p* = 0.563, *BF*_*10*_ = 0.206) nor interaction (*F*(1, 31) = 0.83, *p* = 0.37, *BF*_*10*_ = 0.258) were found.

### Experiment 4

#### Methods

**Figure 4:**
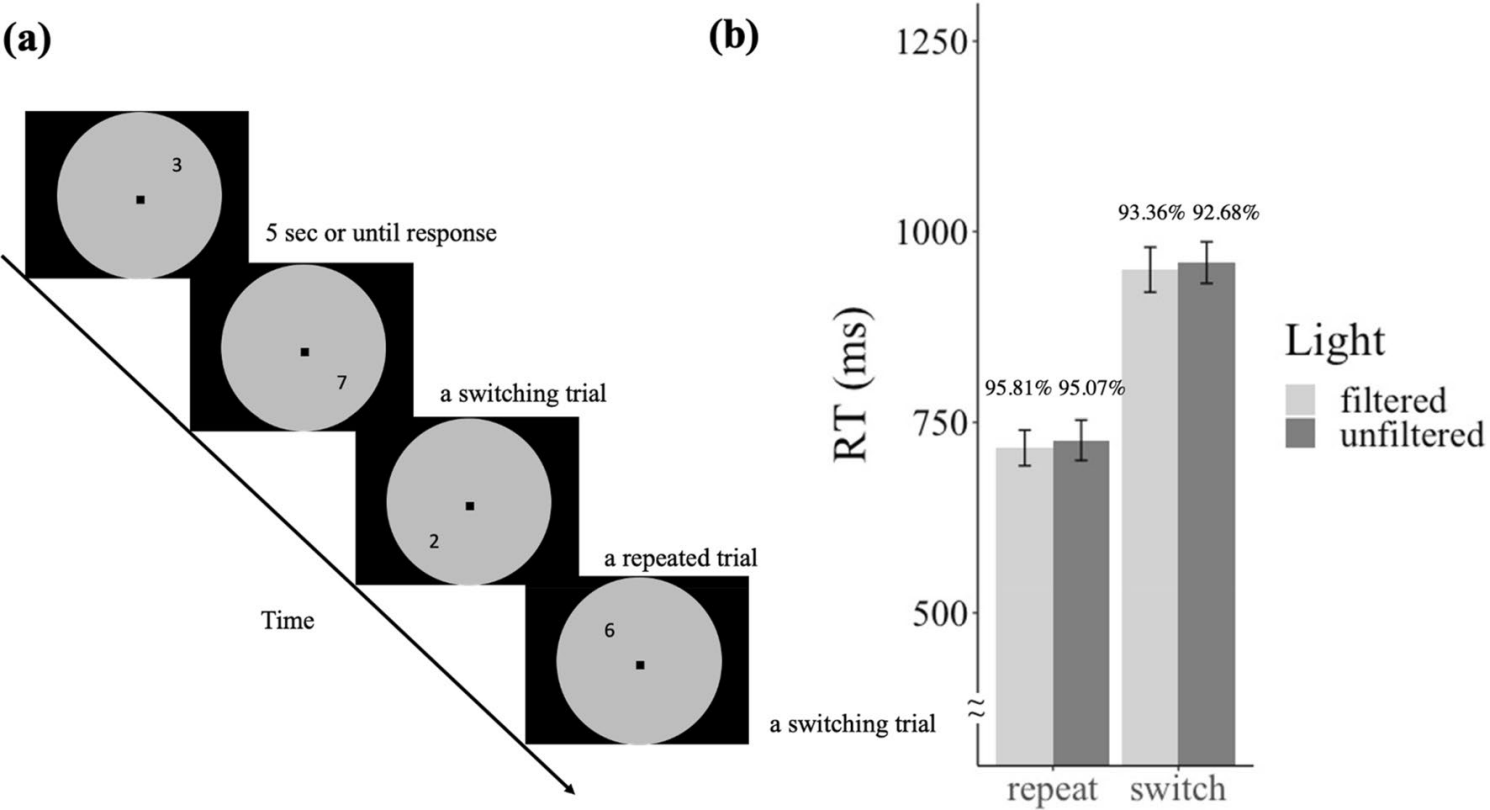
(**a**) The experimental design of Experiment 4. (**b**) Reaction time in repeated and switching trials in Experiment 4. Error bars represent one S.E.M. The number shown above each bar denotes the accuracy of each condition.

Another group of 39 participants were recruited in the current experiment. Data of one participant was excluded due to low accuracy (< 70%). Data of the remaining 38 participants (age range: 20–33, 19 males) was analyzed in the same approach as Experiment 3. The experimental details were the same as in Experiment 3 except for the background colors and the target color. The background color settings were manipulated in the same way as Experiment 2. The number stimuli and the central square (fixation) in Experiment 4 were presented in black. The experimental design is shown in Fig. 4a.

#### Results

For the analysis of RT, data processing was conducted in the same manner as in Experiment 3. Trials that were excluded from analysis consisted of 12.77%. A two-way repeated-measure ANOVA was conducted on the factors of Switch (switching trials, repeated trials) and Light (filtered, unfiltered). Figure 4b shows the results. A significant main effect of Switch was found, *F*(1, 37) = 342.9, *p* < 0.001, *BF*_*10*_ = 3.34 × 10^14^, where the RT for repeated trials were faster than the RT in switching trials. However, no effect of Light (*F*(1, 37) = 0.25, *p* = 0.621, *BF*_*10*_ = 0.174) nor interaction (*F*(1, 37) = 0.002, *p* = 0.963, *BF*_*10*_ = 0.165) between Switch and Light were found. The switch cost was not different across light conditions (*t*(37) = − 0.05, *p* = 0.963, *BF*_*10*_ = 0.175).

As for the accuracy, the overall accuracy was high (94.23%). A higher accuracy was found in the repeated trials compared to the one in switching trials (*F*(1, 37) = 33.26, *p* < 0.001, *BF*_*10*_ = 3.22 × 10^5^). Neither the main effect of Light (*F*(1, 37) = 2.41, *p* = 0.129, *BF*_*10*_ = 0.417) nor interaction (*F*(1, 37) = 0.01, *p* = 0.922, *BF*_*10*_ = 0.295) were found in the accuracy.

#### Discussion

Switch costs were found to be similar across blue and orange light conditions as well as filtered and unfiltered conditions, indicating that cognitive flexibility was not enhanced nor impaired by short time exposure to blue light or ipRGCs. Contradictory to the findings in Ferlazzo et al.^15^ and Slama et al.^16^, we provided null findings regarding blue-light effects on cognitive flexibility measured by the alternating-procedure task-switching paradigm. Given that their studies were confounded with differences in luminance and limited to the afternoon dip session, no conclusive argument could be reached based on their findings. In the current study, we further provided Bayesian statistical values to verify our findings that favored the null hypotheses (i.e., the Bayesian Factors < 0.3). In light of the findings here, the argument indicating that cognitive flexibility under blue light is enhanced should be reconsidered.

The results of Studies 1 and 2 showed that we did not find blue light enhancement effects on the inhibition of irrelevant thoughts and cognitive flexibility using a computer monitor as the light source. It is possible that using a computer monitor as the light source might not yield the same results as the previous studies^13,15^ that showed enhancement in cognitive control. Thus, we next aimed to test if cognitive control focusing on the inhibition of irrelevant visual features can be enhanced under blue light using environmental light. Additionally, Derrfuss et al.^26^ have shown that both the Stroop task and the task switching paradigm share the neural mechanism in the inferior frontal junction, which suggests that the two tasks require similar cognitive functions during the task. Therefore, we selected the Stroop task to examine if blue light influences cognitive control while using an environmental light source in Study 3.

## Study 3

In this study, we investigated whether or not inhibitory control focusing on the suppression of irrelevant visual features would be influenced by blue light. Unlike the previous four experiments, here we used environmental light as the light source. Despite that several studies used the computer monitor to manipulate the light exposure and gained significant effects of blue light on perceptual tasks (e.g., Chen and Yeh^30^, Lee and Yeh^36^, and Yang et al.^38^), the studies that provided evidence of enhancement of blue light in the functions of cognitive control used environmental light^13,15^. Therefore, we used LED light (either blue or green) in Study 3 while asking participants to focus on the numerical size (Experiment 5) or physical size (Experiment 6) in a number-size Stroop task^23,39^. The numerical-size task was more difficult than the physical size one, given that the representation of physical size is more automatic and inhibiting the physical size is more difficult than inhibiting the numerical size^23^. Therefore, the numerical-size task, which required participants to inhibit the representation of the physical size of the number, has a higher cognitive load compared to the physical-size task. By means of examining the Stroop effect in the two tasks, we could further investigate if blue-light effects on inhibitory control depended on cognitive load. The Stroop effect served as the dependent variable and was calculated as the subtraction of the RT of the congruent trials from the RT of the incongruent trials (see “Methods”). Observing smaller Stroop effects in the blue light condition compared to the green light condition would indicate that blue light enhances inhibitory control. Otherwise, no differences across Stroop effects should be found between light conditions.

### Experiment 5

#### Methods

##### Participants

A new group of 16 participants (age range: 20–35, 5 males) were recruited in the current experiment. Other criteria were the same as Studies 1 and 2.

##### Apparatus and stimuli

The target numbers were presented in white (87.1 cd/m^2^) against a gray background (47.2 cd/m^2^) on a i-TECH 20′′ CRT monitor. Participants were instructed to place their head on a chin-rest, which was 85 cm from the monitor. There were two physical sizes of the number, either extending 2.62° or 1.85°. In each trial, two numbers would be randomly chosen (from 1 to 9) and presented 4.5° to the left and right side of the center. The light conditions were manipulated through the LED light tubes with the spectrum 480–485 nm (blue light, power = 12 pcs) and 560–565 nm (green light, power = 12 pcs), suspended 190 cm from the ground (approximately 70 cm from the seated participant’s head).

##### Design and procedure

**Figure 5:**
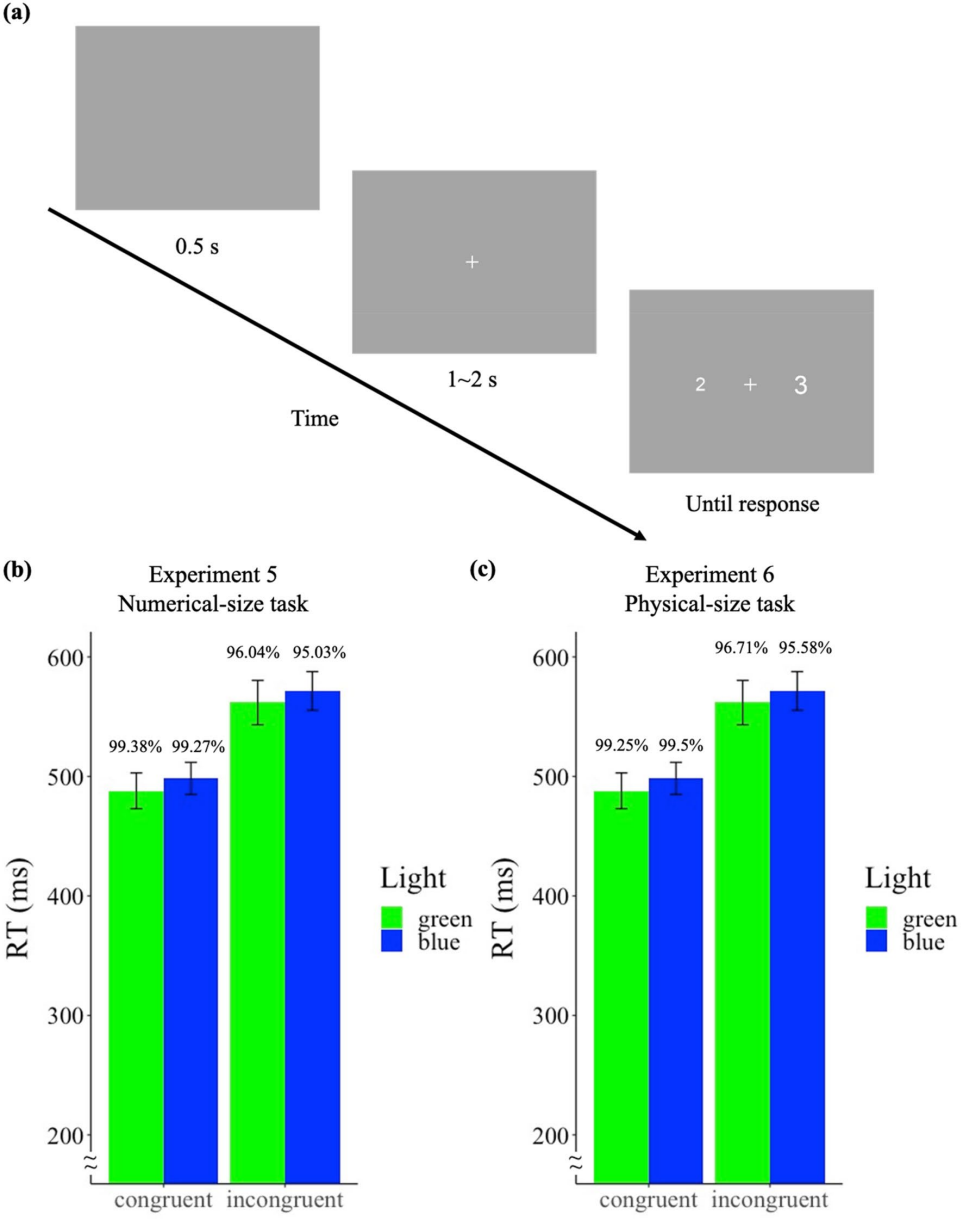
(**a**) The experimental design of Study 3 (note the displays are not to scale). (**b**) Reaction time in congruent and incongruent trials across green and blue light conditions in Experiment 5 (numerical-size task). (**c**) Reaction time in congruent and incongruent trials across green and blue light conditions in Experiment 6 (physical-size task). The number shown above each bar denotes the accuracy of each condition. Error bars represent one S.E.M.

Participants came to the lab for two consecutive days at the same times to avoid possible carryover effects of light. Participants underwent a 5-min environmental light adaptation period before the experiment to equate their experiences towards light exposure and also to stimulate the sluggish ipRGCs^40^. Half of the participants were exposed to the blue environmental light on the first day and green environmental light on the second day, and the reversed order was administered to the other half of the participants. After light adaptation, participants were instructed to conduct 16 practice trials (one block). In this task, two numbers from 1 to 9 were randomly chosen and presented on the left and right visual field. Participants were instructed to judge which side has the number with the larger numerical value by pressing the left or right arrow buttons while ignoring the physical size of the stimuli. The relative numerical value could be in the same direction of the relative physical size (congruent trials, e.g., 2 vs. 3), or be in the opposite direction (incongruent trials, e.g., 2 vs. 3). Figure 5a shows the experimental design. Each trial began with a blank screen for 500 ms, followed by a white fixation (extending 0.96° vertically and horizontally at the center) jittering from 1000 to 2000 ms, and the target numbers would show up on the left and right sides of the fixation until response. Participants were instructed to respond as quickly and accurately as possible. For each light condition, there were 80 trials in a block and three blocks (240 trials) in total, where half of the trials were congruent trials and the other half were incongruent trials. Participants could take a break between the blocks. Participants usually finished one type of light exposure condition in around 30 min.

#### Results

For the analysis of RT, we removed outlier trials (RTs over 3 standard deviations) and the RTs of incorrect trials. Of the trials, 2.94% were excluded from analysis. Figure 5b shows the results. A two-way repeated-measure ANOVA was conducted on the factors of Congruency (congruent trials, incongruent trials) and Light (blue, green). A significant main effect of congruency was found, *F*(1, 15) = 118.1, *p* < 0.001, *BF*_*10*_ = 3.55 × 10^8^, where RTs to congruent trials were faster than those to incongruent trials, suggesting a robust Stroop effect. However, no effect of Light (*F*(1, 15) = 0.69, *p* = 0.419, *BF*_*10*_ = 0.4) nor interaction (*F*(1, 15) = 0.01, *p* = 0.931, *BF*_*10*_ = 0.407) between Congruency and Light were found. The Stroop effect was not different across light conditions (*t*(15) = − 0.09, *p* = 0.931, *BF*_*10*_ = 0.256).

As for the accuracy, the overall accuracy was high (97.58%). A significant higher accuracy was found in the congruent trials compared to the one in the incongruent trials (*F*(1, 15) = 13.22, *p* = 0.002, *BF*_*10*_ = 3.36 × 10^5^). Neither the main effect of Light (*F*(1, 15) = 1.09, *p* = 0.312, *BF*_*10*_ = 0.348) nor interaction (*F*(1, 15) = 0.53, *p* = 0.485, *BF*_*10*_ = 0.638) were found in the accuracy.

### Experiment 6

#### Methods

A new group of 20 participants were recruited in the current experiment (age range: 20–35, 20 males). The experimental details were the same in Experiment 5 except for the instructions. Participants were instructed to judge which side contained the number had a larger physical size by pressing the left or right arrow buttons while ignoring the numerical value of the stimuli.

#### Results

For the analysis of RT, the same data processing as Experiment 5 was conducted. Of the trials, 2.39% were excluded from the analysis. A two-way repeated-measure ANOVA was conducted on the factors of Congruency (congruent trials, incongruent trials) and Light (blue, green). Figure 5c shows the results. A significant main effect of Congruency was found, *F*(1, 19) = 43.87, *p* < 0.001, *BF*_*10*_ = 7.83 × 10^3^, where the RTs to congruent trials were faster than those to incongruent trials, again, suggesting a robust Stroop effect. However, no effect of Light (*F*(1, 19) = 0.11, *p* = 0.747, *BF*_*10*_ = 0.218) nor interaction (*F*(1, 19) = 0.55, *p* = 0.469, *BF*_*10*_ = 0.254) between Congruency and Light were found. The Stroop effect was not different across light conditions (*t*(19) = 0.74, *p* = 0.469, *BF*_*10*_ = 0.273).

As for the accuracy, the overall accuracy was high (97.76%). A significant higher accuracy was found in the congruent trials compared to the incongruent trials (*F*(1, 19) = 17.56, *p* < 0.001, *BF*_*10*_ = 3.37 × 10^5^). No effect of Light was found (*F*(1, 19) = 2.64, *p* = 0.121, *BF*_*10*_ = 0.367). A significant interaction between Light and Congruency was found (*F*(1, 19) = 4.91, *p* = 0.039, *BF*_*10*_ = 0.708). The accuracy of incongruent trials under blue light (95.58%) was slightly lower than the accuracy under green light (96.71%) (*t*(19)  = − 2.09, *p* = 0.1, under Bonferroni correction, *BF*_*10*_ = 1.383), and no color difference was found in the congruent condition (*t*(19) = 1.14, *p* = 0.534, under Bonferroni correction, *BF*_*10*_ = 0.412). Given the relatively high accuracy in the Stroop task and the inconclusive Bayesian Factor (1.383), we did not consider this as a facilitatory effect nor impairment caused by the blue or green light.

In addition, a significant greater Stroop effect was found in Experiment 5 (numerical-size task) compared to Experiment 6 (physical-size task), *t*(34) = 8.23, *p* < 0.001, *BF*_*10*_ = *BF*_*10*_ = 3.15 × 10^6^, suggesting that the cognitive load, as hypothesized, is greater for the numerical-size task than for the physical-size task. Yet, blue light did not cause enhancement nor impairment in the both Stroop tasks.

#### Discussion

Stroop effects were not different in either the numerical-size task or the physical-size task under blue compared to green environmental light. Here we replicated the classic Stroop effect and showed that the numerical-size task was more difficult than the physical-size task^23^. Yet, our results suggest that inhibitory control of irrelevant visual stimuli was not enhanced under blue light. Furthermore, the effect of blue light was not dependent on cognitive load, as the numerical-size task has higher cognitive load compared to the physical-size task^23^. Similar results were found in studies by Tonetti and Natale^41^ and Studer et al.^42^. The authors used blue light versus no light^41^ and blue-enriched light versus red-enriched light^42^ conditions to measure the blue-light effect on cognitive interference. By adopting the attention-network task (ANT)^43^, Tonetti and Natale^41^ and Studer et al.^42^ did not find facilitation in the executive control score (which was calculated as the difference in RT to the incongruent flanker trials and the RT to the congruent flanker trials, as an index of inhibitory control) across lights.

## General discussion

In the three studies, we examined whether blue light influences cognitive control through three different tasks. Despite replicating classic findings in the SART (faster RTs and larger RTCV prior to the no-go target in the mind-wandering condition compared to focused condition), task-switching paradigm (switch cost: faster RTs in the repeated trials compared to the switching trials), and Stroop task (Stroop effect: faster RT in the congruent trials compared to the incongruent trials), none of these experiments showed blue light enhancement effects on cognitive control. Furthermore, we provided Bayesian Factors in each statistical analysis to verify our results. Across the six experiments, we found no Bayesian Factors supporting the facilitation nor impairment of blue light on cognitive control.

Despite that there were slight differences in the stimulation of M cones across conditions in Experiments 1 and 3 (Table 1), the differences can be considered as negligible when compared to the stimulation levels of S cones and ipRGCs. The stimulation levels in M cones were 2.03 cd/m^2^ in the blue light condition and 0.46 cd/m^2^ in the orange light condition, which were very low compared to stimulation levels of S cones (blue: 42.64 cd/m^2^, orange: 0.15 cd/m^2^) and ipRGCs (blue: 19.43 cd/m^2^, orange: 0.28 cd/m^2^). With respect to Experiments 2 and 4, we directly manipulated the stimulation level of ipRGCs (1.43 times higher in the unfiltered condition) using the filter lens, while the stimulation levels in L cones and M cones stayed constant across the blue-light filtered and unfiltered conditions. However, comparisons across light conditions did not reveal any facilitation in cognitive control under blue light resulting from S cones and ipRGCs.

Stronger brain activations might not indicate better behavioral performance. The mPFC^44^, ACC^45^, and IFC^14,46^ are critical brain regions for cognitive control and were found to have stronger activations under blue light^3–6,47,48^. Furthermore, Hung et al.^49^ also found that the stimulation of ipRGCs will activate the frontal eye field, which is also a critical brain region for inhibitory control^50^. Even if stronger activations in these brain regions related to cognitive control were found under blue light, no behavioral enhancement was found in the current study and most of the studies mentioned above, with the exception of Daneault et al.^5^. Although the modulation of brain activity usually results in change of behaviors, this might not be the case for effects of blue light on cognitive control. According to the global neuronal workspace theory^7,8^, our results indicate that brain activations induced by blue light might not be strong enough to drive differences in behavioral responses, at least in the domain of cognitive control. Instead, the change of activity brought by blue light might be limited to the neural level. Future studies can correlate the enhancement in these brain regions induced by blue light with behavioral performance to directly examine if these enhanced activations induced by blue light are related to the performance of cognitive control.

Previous studies have shown blue-light enhancement in cognitive control, but in those studies, several factors are not well-controlled or are missing. For example, Ferlazzo et al.^15^ and Slama et al.^16^ found smaller switch costs under blue light compared to halogen and orange light, respectively. However, the present study, with careful control of equivalent luminance and manipulation of activations in S cones and ipRGCs (Table 1), failed to find the blue-light enhancement on switch cost. It is likely that the results showing smaller switch costs under blue light were caused by inadequate controls, as the control light would have stimulated various types of photoreceptors at different levels, especially with differences in luminance. In addition, although we attempted to use analogous experimental settings in our Study 3, namely, using environmental light conditions instead of light emitted by the monitor as previous studies showed gains of blue light on cognitive control, we found no facilitation effect of blue light on the Stroop task under both high and low cognitive loads. The caveat (i.e., inadequate controls) also applies to the studies conducted by Killgore et al.^51^ and Alkozei et al.^52^, which were studies conducted by the same research group. Killgore and his colleagues proposed that blue light would enhance neural efficiency in resolving cognitive interference^51^ and decrease the reaction time in the n-back working memory task^52^. However, their experimental procedures were very different from other studies. For example, they adopted a between-subjects design instead of a within-subjects design that most studies used. Namely, in these two studies^51,52^, different groups of participants were exposed to blue-light and amber-light. It is thus likely that the observed differences in results under different lights were due to between-subject differences, as Killgore et al.^51^ found no behavioral differences whereas Alkozei et al.^52^ found one despite their identical experimental settings and procedures. In addition, participants in Killgore et al.^51^ and Alkozei et al.^52^ both experienced a “wash-out” session before the light exposure phase, where both groups of participants were exposed to amber light prior to the formal light exposure phase. Therefore, the extra exposure to the amber light for the amber light group could be an alternative explanation for their findings that blue light enhanced, or the amber light impaired, human cognitive control. All in all, we regard the differences in experimental designs and procedures are the main reasons for the inconsistencies across studies. Future studies should carefully manipulate and control these parameters to investigate the effects of blue light on human cognition.

An alternative explanation is that previous studies observing the color difference in performance did not result from the influence of blue light per se, but resulted from the control light impairing performance. For example, Chien et al.^35^ showed a shift of the point of subjective simultaneity (PSS) during an audiovisual simultaneity task to a visual-leading condition under red light exposure compared to blue light exposure in their Experiment 1. Furthermore, as they controlled for the activation of ipRGCs in their Experiment 2, the shift of PSS vanished in the ipRGC-high versus ipRGC-low conditions, showing no support for ipRGC mediated blue-light effects on the PSS. Specifically, the speed of visual processing was modulated by the inhibition of the magnocellular system under red light, rather than the effects of blue light. Since most studies did not systematically manipulate the stimulation level of each photoreceptor, we cannot conclusively attribute their effects to the control light(s) that they used. We propose that this could be a possible reason for the inconsistencies in previous studies as light conditions varied greatly, so the cause of any effects could potentially be attributed to blue light or the control light. As for the current study, null results were found even though we provided similar luminance across blue and orange light and carefully manipulated the stimulation levels of ipRGCs.

In the modern generation, we are frequently exposed to high-dosages of blue light in the office or at home. However, based on the results of the current study, exposure to blue light or the stimulation of ipRGCs will neither enhance nor impair the performance of cognitive control, at least in the conditions we investigated. Even though our previous study^36^ has shown that blue light can enhance saccadic eye movements and attentional disengagement, Lee and Yeh^36^ mainly focused on the facilitation of saccade latency rather than how blue light affects information processing after the saccadic eye movement. Namely, to saccade to the target faster is not equivalent to processing the target more efficiently: whether blue light influences processing efficacy hinges on whether blue light influences cognitive control. From the current study, we suggest that blue light neither enhances nor impairs cognitive control, and so the use of technology might not cause impairments to cognition as people might typically think.

In conclusion, despite the close relationship of working memory, inhibition, and cognitive flexibility in cognitive control, effects of blue light on working memory (if any) might not parallel with cognitive flexibility and inhibitory control. The current study provided the first systematic investigation of blue-light effects on cognitive control with well-established control of light parameters. The issue of whether the enhancement of blue light on brain activities could be generalized to the behavioral level should be carefully re-examined.

## Data Availability

The datasets generated and analyzed during the current study are available in https://osf.io/v2apc/?view_only=bb88e03fbbc14bf0995a9fa5f082871d.
